# Ear Mite Removal in the Santa Catalina Island Fox (*Urocyon littoralis catalinae*): Controlling Risk Factors for Cancer Development

**DOI:** 10.1371/journal.pone.0144271

**Published:** 2015-12-07

**Authors:** Megan E. Moriarty, T. Winston Vickers, Deana L. Clifford, David K. Garcelon, Patricia M. Gaffney, Kenneth W. Lee, Julie L. King, Calvin L. Duncan, Walter M. Boyce

**Affiliations:** 1 Karen C. Drayer Wildlife Health Center, School of Veterinary Medicine, University of California Davis, Davis, California, United States of America; 2 Institute for Wildlife Studies, Arcata, California, United States of America; 3 Wildlife Investigations Laboratory, California Department of Fish and Wildlife, Rancho Cordova, California, United States of America; 4 Department of Pathology, Microbiology, and Immunology, School of Veterinary Medicine, University of California Davis, Davis, California, United States of America; 5 Departments of Pathology and Medicine, University of California San Diego, San Diego, California, United States of America; 6 Greer Laboratory, Lenoir, North Carolina, United States of America; 7 Catalina Island Conservancy, Avalon, California, United States of America; Colorado State University, UNITED STATES

## Abstract

Ear mites (*Otodectes cynotis*) and ear canal tumors are highly prevalent among federally endangered Island foxes (*Urocyon littoralis catalinae*) living on Santa Catalina Island off the coast of Southern California. Since studies began in the 1990s, nearly all foxes examined were found to be infected with ear mites, and ceruminous gland tumors (carcinomas and adenomas) were detected in approximately half of all foxes ≥ 4 years of age. We hypothesized that reduction of ear mite infection would reduce otitis externa and ceruminous gland hyperplasia, a risk factor for tumor development. In this study, we conducted a randomized field trial to assess the impact of acaricide treatment on ear mite prevalence and intensity of infection, otitis externa, ceruminous gland hyperplasia, and mite-specific IgG and IgE antibody levels. Treatment was highly effective at eliminating mites and reducing otitis externa and ceruminous gland hyperplasia, and mite-specific IgG antibody levels were significantly lower among uninfected foxes. Ceruminous gland hyperplasia increased in the chronically infected, untreated foxes during the six month study. Our results provide compelling evidence that acaricide treatment is an effective means of reducing ear mites, and that mite removal in turn reduces ear lesions and mite-specific IgG antibody levels in Santa Catalina Island foxes. This study has advanced our understanding of the underlying pathogenesis which results in ceruminous gland tumors, and has helped inform management decisions that impact species conservation.

## Introduction

Disease is a significant threat to conservation-dependent wildlife species, particularly for genetically and geographically isolated populations [1]. The Santa Catalina Island (SCA) fox (*Urocyon littoralis catalinae*) is one of six distinct subspecies [2,3] native to the Channel Islands off the coast of Southern California (SCA: 33°24’ N, 118°24’ W). In 1999, a suspected distemper epidemic decimated 85–90% of this population, and the remaining ~100 foxes in this subspecies were listed as federally endangered [4,5]. Intensive management actions including translocation, captive breeding, and vaccination successfully averted extinction and contributed to rapid population growth to an estimated population of 1,114 in 2012 [6, 7]. Although the population is now increasing, disease epidemics remain a primary threat to the continued existence of the SCA foxes [8, 9].

**Fig 1:**
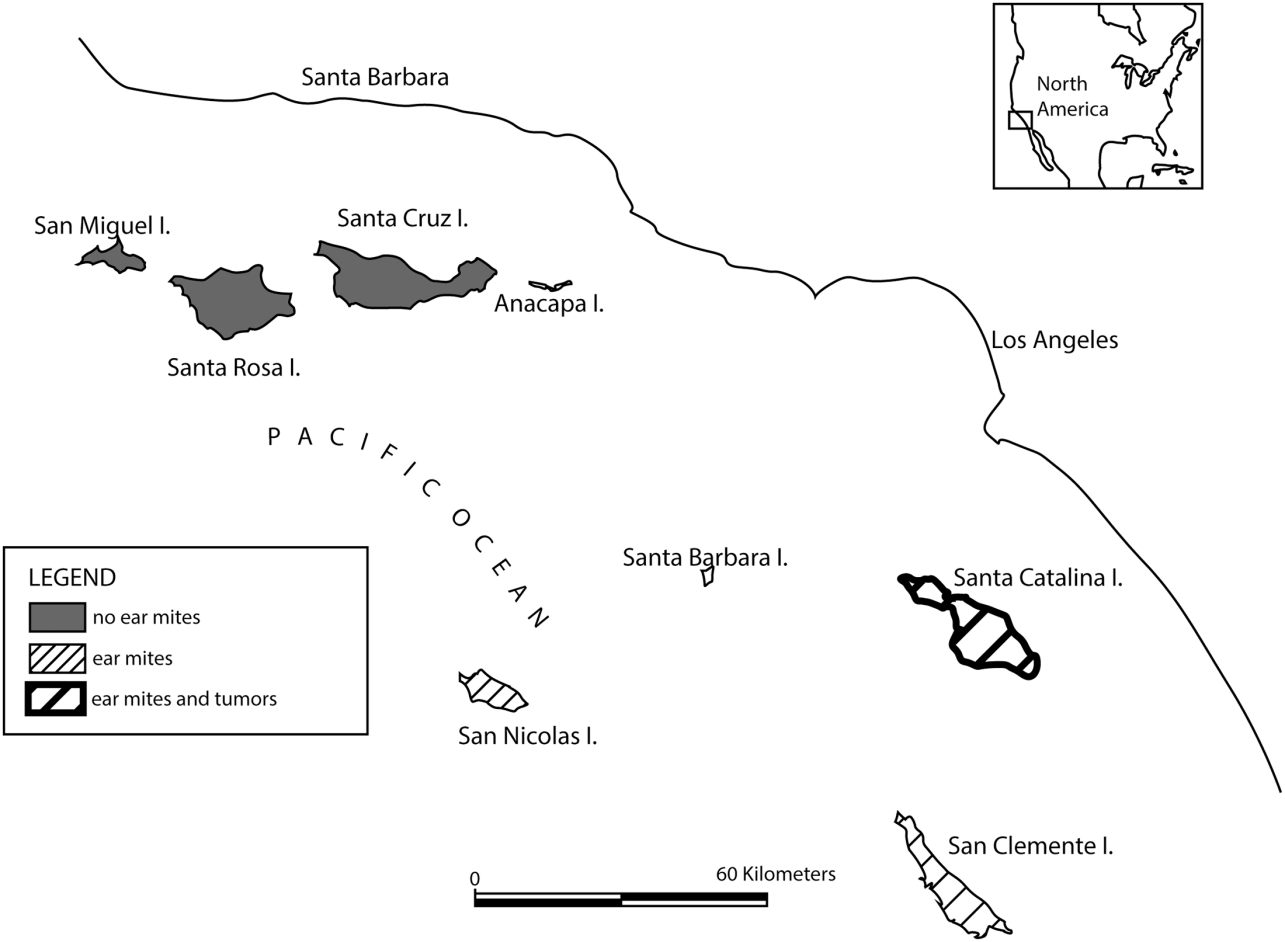
Map of the Channel Islands (California, USA). Distribution of foxes that are uninfected or infected with ear mites, and foxes that have ear canal tumors. Island foxes do not reside on Santa Barbara or Anacapa islands.

Ceruminous gland tumors (carcinomas and adenomas) were first observed in SCA foxes in 2001 [10]. In our companion study conducted from 2001–2008, Vickers et al. found that approximately half of SCA foxes ≥ 4 years of age had ceruminous gland tumors, and that ear mites were present in > 98% of SCA foxes [10]. Ear mites have been detected in two other subspecies of Channel Island foxes in the Southern islands, but never in the three subspecies in the Northern islands; however, SCA foxes are the only subspecies that have ear canal tumors (Fig 1) [8,10]. It remains unclear why some subspecies of island foxes have ear mites and others do not, but potential hypotheses include historical introduction of ear mites by feral cats to the Southern islands [8], as well as possible differences in immune response or genetic susceptibility. Quantitative risk assessment showed that tumors are associated with ceruminous gland hyperplasia (CGH), which is in turn strongly correlated with otitis externa caused by ear mites (*Otodectes cynotis*) [10]. These findings are consistent with the hypothesis that SCA foxes are uniquely predisposed to severe ear canal lesions, and that ear mites incite an inflammatory response and ceruminous gland hyperplasia that precede and may promote tumor formation and carcinogenesis [10].

Ear mites live on and in the superficial keratin of the ear canal and cause an inflammatory reaction in the dermis that increases cerumen production and keratin debris, sometimes obstructing the ear canal, and leading to ceruminous gland hyperplasia [11–13]. This thickened ceruminous exudate can be carcinogenic [14], and a study in dogs and cats demonstrated a strong association between chronic otitis externa and the development of ceruminuous gland hyperplasia and neoplasia [15]. In SCA foxes, mite infection has been seen in association with marked otitis externa, peri-aural wounds, cellulitis, localized secondary bacterial infections and septicemia, as well as ceruminous gland carcinomas with local invasion and distant metastasis [8,10]. Although mites appear to initiate a cascade of events that may predispose to ceruminous gland tumors, no previous studies have examined what effect mite removal could have on disease progression.

The objective of this randomized six month field trial was to assess the effect of acaricide treatment on ear mites, ear canal lesions, and levels of mite-specific antibodies (IgG and IgE) in free-ranging SCA foxes. In addition, we compared levels of mite-specific antibodies from SCA foxes with Santa Cruz Island (SCZ) foxes (*Urocyon littoralis santacruzae*), a closely related subspecies with no evidence of ear mites, otitis, or ceruminous tumors. We hypothesized that (1) mite prevalence and intensity of infection would be significantly reduced by treating foxes with acaricide; (2) mite removal would be associated with reduced otitis and ceruminous gland hyperplasia; and (3) mite removal would be associated with reduced levels of mite-specific IgG and IgE.

## Materials and Methods

### Ethics statement

Animal capture and biological sampling of SCA foxes was authorized under a U.S. Fish and Wildlife Service (USFWS) Federal 10(a)1(A) permit (No. TE744878-9) and a Memorandum of Understanding with the California Department of Fish and Wildlife (CDFW). All procedures involving animals were reviewed and approved by the University of California, Davis, Institutional Animal Care and Use Committee (Protocol No. 13366).

### Field Trial Design

SCA foxes were captured in July 2009 (t_0_), September 2009 (t_1_), November 2009 (t_2_), and January 2010 (t_3_) (Table 1). Foxes captured at t_0_ were included in the study if they had mites on initial exam, were greater than 1 year old, had not been treated for ear mites in the past 12 months, and had no history of previous lateral wall resection of the ear canal for tumor removal. Enrolled foxes were randomly assigned to the treated or untreated comparison groups in blocks of two, which occurred at time of capture and followed *a priori* instructions to randomly assign the first fox to one of the groups, and alternate group allocation thereafter.

**Table 1:** Field trial design and samples collected from treated and untreated Island foxes at each capture period.

	t_0_ (July 2009)	t_1_ (Sept. 2009)	t_2_ (Nov. 2009)	t_3_ (Jan. 2010)
Ear mite assessment^1^	X	X	X	X
Ear pathology assessment^2^	X			X
IgG and IgE assessment^3^	X	X	X	X
Acaricide treatment^4^	X^a^	X^a^	X^a^	X^b^

^1^ Ear mite score and count, as determined by otoscopic and microscopic examination, respectively

^2^ Otitis and ceruminous gland hyperplasia scores, as determined by histopathology

^3^ Serum mite-specific IgG and IgE levels, as determined by immunoassays

^4^ Acaricide treatment included ivermectin aurally and fipronil topically

^a^ Only the treated group

^b^ Both treated and untreated groups

Box traps (Tomahawk Live Trap Company, Tomahawk, Wisconsin, USA) were set and baited with a combination of dog kibble, canned cat food, and berry paste. Traps were placed along transects near roads and trails, at 250–300 meter intervals. One to two traps were set at each site, with 15–44 sites used each day. All traps were checked each morning, and foxes were examined, sampled, treated, and released at the site of capture. Foxes were blindfolded and manually restrained while the project veterinarian (TWV) performed a physical examination and otoscopic assessment of both ear canals. Baseline data were collected, including passive integrated transponder tag number, sex, age, weight, physical condition, and ear canal observations. Foxes 1–3 years old were classified as young and foxes ≥ 4 years old were classified as mature. If exact age could not be determined from previous capture history, foxes were assigned to age groups using tooth wear patterns [16,17], and foxes were categorized as young if they had Class 1 dentin exposure patterns or mature if they had Class 2–4 dentin exposure patterns.

Ear mite infection intensity was assessed by two methods: mite score and mite count. A subjective mite severity score was determined otoscopically by the project veterinarian (Table 2). After scoring, a cotton swab was inserted into each ear canal to collect mites, then placed in a glass tube and kept in an insulated container with ice packs until being stored at 4–8°C. Within a week of collection, samples were shipped on dry ice to the laboratory at the University of California, Davis, and the number of mites was determined using a dissecting microscope (MEM). Both mite score and mite count were averaged between left and right ears to provide an overall estimate of infection intensity.

**Table 2:** Scoring criteria: otoscopic assessment of mites and histopathologic assessment of otitis and ceruminous gland hyperplasia (CGH).

Mite score	
0	No mites
1	Few mites, scattered mites throughout the ear canal
2	One cluster of mites, some scattered mites throughout the ear canal
3	Two clusters of mites, some scattered mites throughout the ear canal
4	Multiple clusters of mites throughout the ear canal
Otitis score	
0	None
1	Few plasma cells, mast cells, neutrophils or eosinophils in the dermis
2	Moderate numbers of plasma cells, lymphocytes, mast cells, neutrophils or eosinophils present in the epidermis and/or expanding the dermis
3	Abundant plasma cells, lymphocytes, mast cells, neutrophils or eosinophils in the epidermis and dermis with destruction of epidermal and dermal components
CGH score	
0	None
1	Mild gland ectasia and piling up of cells within glands
2	Moderate gland ectasia and increased number of glands
3	Marked gland ectasia and adenomatous gland clusters

Ear canal biopsies were performed under local anesthesia with lidocaine 4% gel, and lidocaine injectable solution infused subcutaneously immediately adjacent to the biopsy site. Using a sterile 5–6 mm skin punch, biopsies were taken from the lower caudal aspect of the left and right vertical ear canals at the junction with the horizontal canals, and fixed in 10% neutral buffered formalin. Proliferative lesions were also biopsied and removed entirely if they completely obstructed the canal. After obtaining the sample, digital pressure and styptic powder were applied to the biopsy site to promote hemostasis. A nonsteroidal anti-inflammatory drug (Ketoprofen, 2 mg/kg, Zoetis Inc., Kalamazoo, MI, USA) and systemic antibiotic (Enrofloxacin, 10 mg/kg, Bayer, West Haven, CT, USA) were given subcutaneously.

A 10 ml blood sample was collected by jugular venipuncture into a serum collection tube and kept in an insulated container with ice packs. After clotting, blood samples were refrigerated and centrifuged at 1176 x g (Fischer Scientific Centrific Model 225) within 24 hours of capture; sera were dispensed into aliqots and stored at -80°C until used for testing mite-specific IgG and IgE. Foxes in the treated group received fipronil (Frontline Top Spot, 30 mg/kg, Merial, Ltd., Duluth, GA, USA) topically between the shoulder blades and ivermectin (Acarexx, 0.5 ml of 0.01% solution, Boehringer Ingelheim Vetmedica, Inc., St. Joseph, MO, USA) in each ear. Fipronil was given as an augmentation to assist with treatment of *Otodectes*. This is an off-label use, but was justified to reduce the likelihood of immediate re-infestation from contact with other foxes (longer half-life than ivermectin), and to prevent reintroduction of mites from one ear to the other via the skin surface. Foxes in the untreated comparison group did not receive either treatment.

Efforts to recapture study foxes were made every two months over the six month trial period (t_1_, t_2_, t_3_). At the first two recapture events (t_1_, t_2_), all foxes were examined and sampled (ear mites and blood), while only treated foxes received acaricide (Table 1). At the end of the six month period (t_3_), all foxes were examined, sampled (ear mites and blood), biopsied, and treated to maximize the number of treated foxes in the SCA population at the end of the study (Table 1).

### Histopathology assessment of biopsies

Ear canal biopsies were routinely processed, paraffin-embedded, sectioned at 5 μm and stained with hematoxylin and eosin. Immunohistochemistry for pan-cytokeratin was used for definitive tumor diagnosis. All histology was interpreted by a board-certified pathologist (PMG) at the University of California, Davis, who remained blind to treatment. Specimens were evaluated for evidence of inflammation consistent with otitis (Table 2), CGH (Table 2), and presence or absence of ceruminous gland adenoma or carcinoma. The scores from the left and right ear were averaged to provide an overall estimate of otitis and CGH.

### Mite extracts and preparation of coated wells

Ear mites isolated from SCA foxes during field evaluations were used to prepare aqueous solutions of mite-specific antigens. Briefly, mites were milled using a Tenbroeck tissue grinder then suspended in Phosphate Buffered Saline (PBS). Following overnight incubation at -20°C, the mite suspension was centrifuged at 12,857 x g and the supernatant was collected and used as mite-specific antigen. The protein content was determined colorimetrically with a BCA Protein Assay (Thermo Fisher Scientific, Pierce, Rockford, IL, USA). Immulon 4HBH flat bottom strip assemblies (Thermo Electron Corporation, Waltham, MA, USA) were used throughout and served as the solid phase for all direct bind enzyme-linked immunosorbent assays (ELISA). The 12-well strips were individually coated with mite extracts (1 μg/mL) following a previously defined procedure [18]. Briefly, individual extracts were diluted in bicarbonate buffer (pH 9.6) and 350 μL was added to each assigned well. Following overnight incubation at 4–8°C, the wells were washed with PBS, blocked with 1% monoethanolamine (pH 7.5), air dried and stored at -20°C in Ziplock bags until used.

### ELISA for mite-specific IgG and IgE

The presence of mite-specific IgE antibody in fox serum was assessed using a canine IgE specific ELISA [18, 19]. A similar semi-quantitative ELISA specific for fox IgG was developed for this study in order to determine the relative level of mite-specific IgG antibody in each sera sample (KWL and MEM). For the IgG assay, three-fold serial dilutions of fox sera ranging from 1:150 to 1:36,450 were added, in triplicate, to plates coated with antigens extracted from homogenized ear mites. Biotinylated monoclonal anti-IgG antibodies were used as the primary probe reagent, streptavidin alkaline phosphatase as the enzyme containing secondary tracer, and p-nitrophenylphosphatase as the chromogenic substrate [18].

To standardize the results of the assay and enable consistent comparisons of assay results, across multiple assay runs and variable serum dilutions, all results were normalized to a four point calibration [20]. The relative quantity of mite-specific IgG was interpolated from a four point regression curve created by plotting the background-corrected optical density observed with each of the calibrators versus an arbitrarily assigned concentration value based upon the dilution schema used for preparing the calibrator solutions. The relative IgG level present in the original serum sample is the product of the interpolated value and the serum dilution factor that was tested. Sera collected from Santa Cruz Island (SCZ) foxes (n = 43) served as ear mite uninfected comparisons. The foxes on this island are a separate subspecies and ear mites have never been detected on examination of live or dead SCZ foxes.

### Statistical analysis

To determine whether or not treated and untreated foxes were comparable at the beginning of the study, we compared age, sex, gross ear canal assessment (ear mites, proliferative lesions, inflammation, nodular thickening, pus), histologic ear canal assessment (otitis externa, CGH, sebaceous gland hyperplasia, epidermal hyperplasia, acanthosis, hyperkeratosis, adenoma, carcinoma, or previous tumor diagnosis), and mite-specific IgG/IgE levels using Yates’ corrected chi-square test, two-sample t-test, and Mann-Whitney U test [21]. Recapture success, defined as the proportion of initially sampled foxes resampled at each capture period, was compared with a Pearson’s chi-square test [21].

To determine whether or not ear mite burden decreased after treating foxes with acaricide, we evaluated both mite prevalence and intensity of infection. Prevalence was defined as the proportion of foxes with a mite score and/or count > 0, and uninfected foxes were defined as those with both mite score and count equal to 0. Intensity of infection was assessed using both mite score and count, and a Spearman’s rank test was performed to assess correlation between the two measures [21]. Prevalence and intensity of infection were compared between treated and untreated groups at each capture period using a Pearson’s chi-square test and Mann-Whitney U test, respectively. Changes in prevalence and intensity of infection from t_0_ and t_3_ were evaluated using a McNemar chi-square test and Friedman test, respectively [21].

To determine whether or not otitis and CGH were reduced by mite removal, histopathology scores were compared between treated and untreated groups at t_3_ using a Mann-Whitney U test. Changes in ear pathology between t_0_ and t_3_ were evaluated using a Friedman test. To determine whether IgG and IgE levels were reduced by mite removal, values were compared between treated and untreated groups at each capture period using a Mann-Whitney U test. Changes in IgG and IgE levels between t_0_ and t_3_ were compared using a Friedman test. IgG and IgE levels were compared between SCA foxes at each capture period and SCZ foxes using a Mann-Whitney U test.

Because treatment is simply a method for reducing mites, and mite presence and intensity are the actual risk factors of interest, all of the above analyses were also performed comparing infected and uninfected foxes, irrespective of treatment group. All statistical analyses were performed using SPSS software (version 20.0, IBM SPSS Statistics for Windows, Armonk, NY) with an alpha level of 0.05.

## Results

One hundred seventeen foxes were enrolled in the study: 59 were assigned to the treated group and 58 to the untreated group (S1 Table). During the course of the study, two foxes, both from the treated group, died from vehicle trauma., No adverse effects of sampling or treatment were observed during recapture examinations. At the beginning of the study, no significant differences were found between treated and untreated groups in regards to age, sex, gross ear canal assessment, histologic ear canal assessment, or mite-specific IgG/IgE levels (p ≥ 0.1). Recapture success was similar between treated and untreated foxes at t_1_ (39/59 = 66% and 43/58 = 74%, respectively) and t_3_ (40/59 = 68% and 37/58 = 64%, respectively) (p ≥ 0.3); however, fewer treated foxes were recaptured than untreated foxes at t_2_ (25/59 = 42% and 36/58 = 62%, respectively) (p = 0.03) (S1 and S2 Figs).

### Effect of acaricide on mite prevalence and intensity of infection

**Fig 2:**
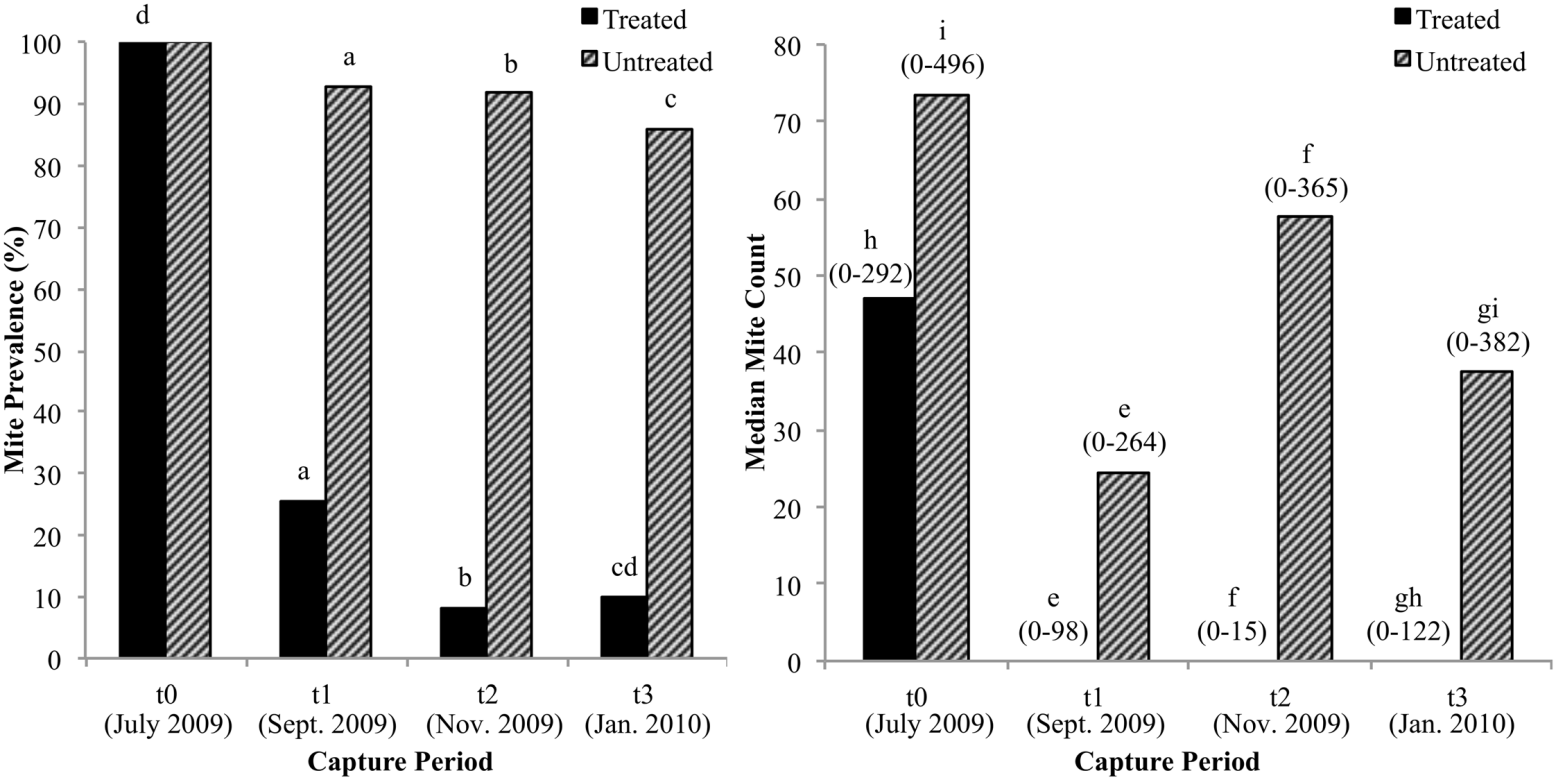
Ear mite prevalence and intensity of infection in treated and untreated Island foxes. Prevalence: number of foxes with mites/total number of foxes. Intensity of infection: median ear mite count. Number of treated and untreated foxes (t_0_: 59, 58; t_1_: 39, 42; t_2_: 24, 36; t_3_: 40, 36). Range of values for mite intensity shown in parentheses above each bar. Foxes excluded from analysis: one untreated fox at t_1_ and t_2_ (missing mite count), one untreated fox at t_3_ (accidentally treated at t_2_). ^a, b, c^ Significant difference (Pearson’s chi-square test, p < 0.001). ^d^ Significant difference (McNemar’s chi-square test, p< 0.001). ^e, f, g^ Significant difference (Mann-Whitney U test, p < 0.001). ^h, i^ Significant difference (Friedman test, p < 0.001, p = 0.02).

Ear mite score and count were significantly correlated (p < 0.001), therefore mite count was used to describe intensity of infection. Treated foxes had significantly lower mite prevalence and intensity of infection when compared with untreated foxes at t_1_, t_2_, and t_3_ (p < 0.001; Fig 2). Mite prevalence declined significantly within the treated group (p < 0.001), but not within the untreated group (p = 0.1) between t_0_ and t_3_. However, mite infection intensity decreased significantly within both the treated (p < 0.001) and untreated groups (p = 0.02) between t_0_ and t_3_. We estimated the duration of acaricide efficacy by examining a subset of foxes that were only treated once (at t_0_), then re-evaluated two, four, or six months later. Among treated foxes, 10/39 were infected two months later, 1/7 were infected four months later, and 3/7 were infected six months later (S1 Fig).

### Effect of mite removal on otitis and hyperplasia

**Fig 3:**
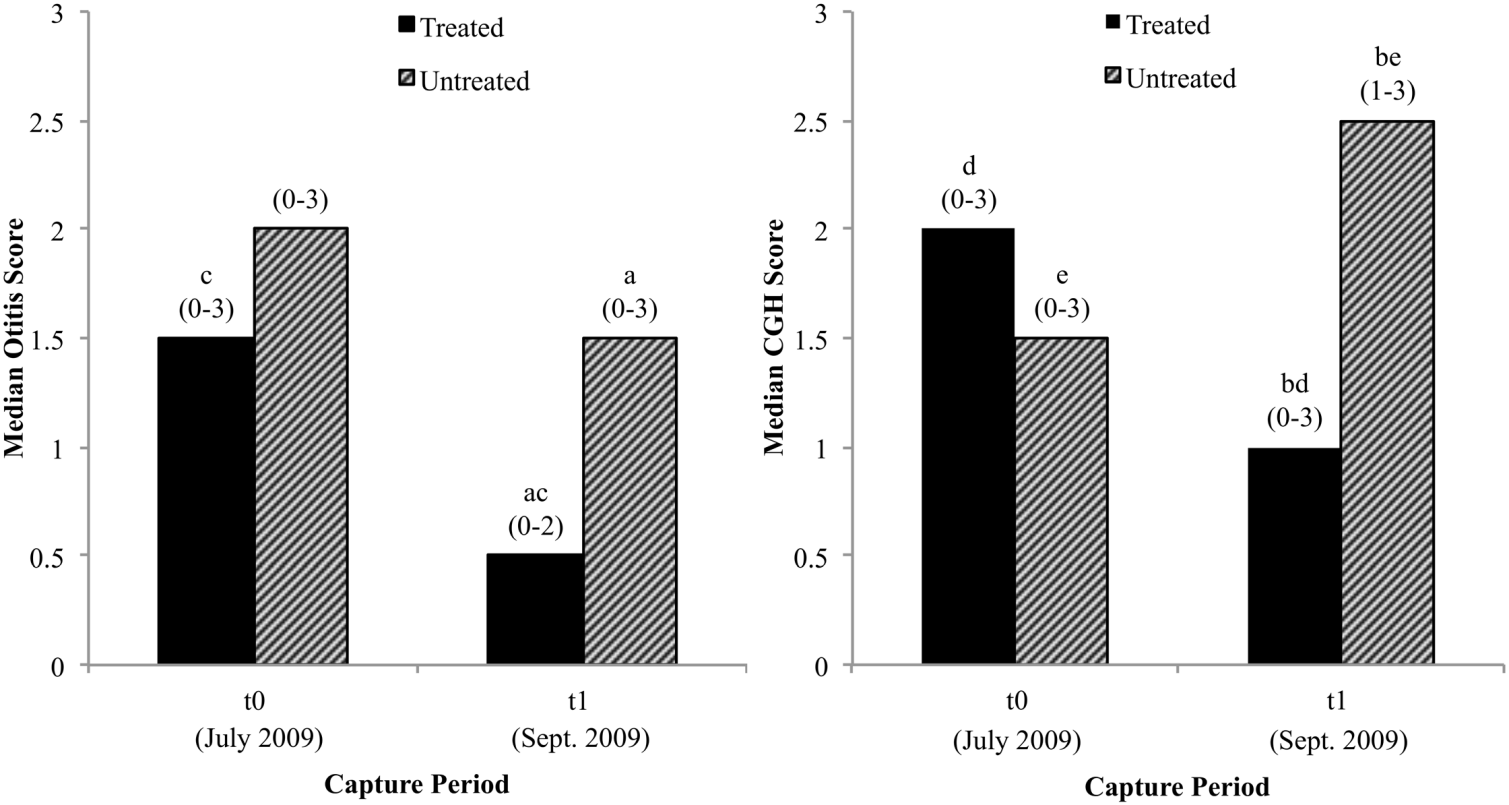
Otitis and ceruminous gland hyperplasia (CGH) scores in a subset of Island foxes recaptured at t_3_. Comparison between treated and untreated foxes at t_3_, and between t_0_ and t_3_ within each group. Number of treated and untreated foxes at t_0_ and t_3_ (37, 33). Range of values for otitis and CGH shown in parentheses above each bar. Foxes excluded from analyses: three treated and two untreated foxes at t_0_, one treated and untreated fox at t_3_ (non-diagnostic biopsy), 18 treated and 22 untreated foxes at t_3_ (not recaptured). ^a,b^ Significant difference (Mann-Whitney U test, p < 0.001). ^c, d, e^ Significant difference (Friedman test, p ≤ 0.007).

Treated foxes exhibited significantly lower otitis and CGH scores than untreated foxes at t_3_ (p < 0.001; Fig 3). Between t_0_ and t_3_, otitis scores declined significantly within the treated group (p < 0.001), while there was no significant change within the untreated group (p = 0.5). CGH scores also significantly decreased within the treated group (p = 0.007) between t_0_ and t_3_, but in contrast to otitis scores, CGH scores significantly increased within the untreated group (p = 0.001) between t_0_ and t_3_. The same findings were observed when uninfected foxes were compared to infected foxes.

### Effect of mite removal on IgG and IgE levels

**Fig 4:**
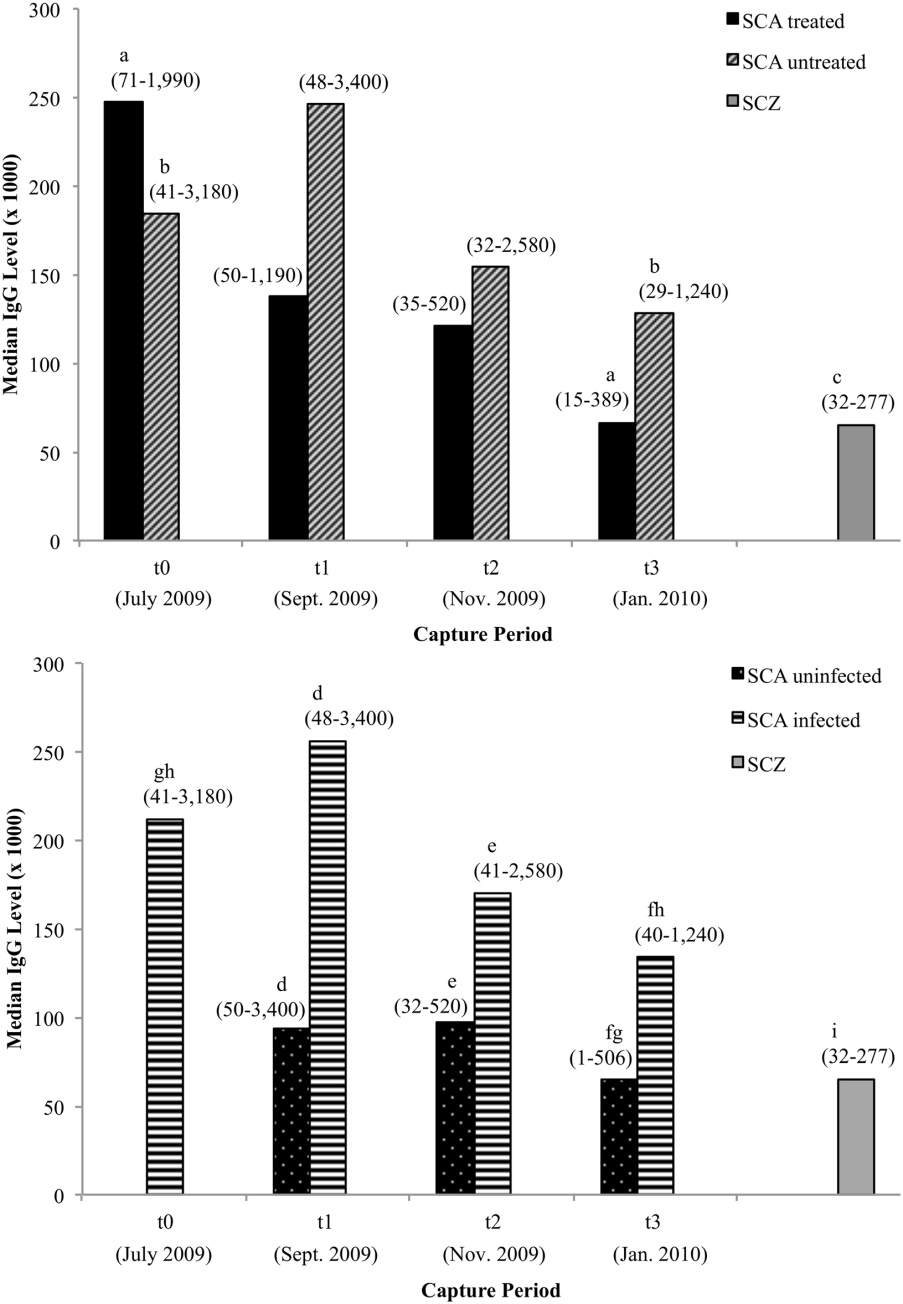
IgG levels in Santa Catalina Island treated/untreated and uninfected/infected foxes, and Santa Cruz Island foxes. IgG level: a relative quantity of mite-specific antibody (determined by interpolation from a four-point calibration curve, see text for further explanation). Number of treated and untreated foxes (t_0_: 59, 57; t_1_: 38, 42; t_2_: 24, 36 t_3_: 40, 36); number of uninfected and infected foxes (t_0_: 116; t_1_: 31, 49; t_2_: 25, 35 t_3_: 42, 35); number of SCZ foxes (43). Range of values for IgG shown in parentheses above each bar. Foxes excluded from analyses: one untreated/infected fox at t_0_ and one treated/uninfected fox at t_1_ (missing IgG level). ^a, b^ Significant difference (Friedman test, p < 0.001). ^c^ Significant difference between SCZ foxes vs. SCA treated and untreated foxes at all capture events (Mann-Whitney U test, p ≤ 0.01), except for SCA treated foxes at t_3_ (p = 0.6). ^d, e, f^ Significant difference (Mann-Whitney U test, p ≤ 0.01). ^g, h^ Significant difference (Friedman test, p ≤ 0.004). ^i^ Significant difference between SCZ foxes vs. SCA infected and uninfected foxes at all capture events (Mann-Whitney U test, p ≤ 0.001), except for SCA uninfected foxes at t_2_ (p = 0.1) and t_3_ (p = 0.9).

Treated and untreated groups did not have significantly different IgG levels at t_1_ (p = 0.08), t_2_ (p = 0.3), or t_3_ (p = 0.06), but there was a significant decrease in IgG levels within both groups between t_0_ and t_3_ (p < 0.001; Fig 4). Both groups of SCA foxes at t_0_ had significantly higher IgG levels than uninfected SCZ foxes (p < 0.001). The SCA treated foxes maintained significantly higher IgG levels than SCZ foxes at t_1_ (p < 0.001) and t_2_ (p = 0.01), but their IgG decreased to levels similar to SCZ foxes at t_3_ (p = 0.6). In contrast, SCA untreated foxes maintained significantly higher IgG levels than SCZ foxes at all recapture periods (t_1_ and t_2_: p < 0.001, t_3_: p = 0.004).

When IgG levels among SCA foxes were compared based on infection status, uninfected foxes had significantly lower IgG levels than infected foxes at t_1_ (p < 0.001), t_2_ (p = 0.01), and t_3_ (p = 0.01; Fig 4). Between t_0_ and t_3_, there was a significant decrease in IgG levels within both uninfected and infected foxes (p < 0.001, 0.004). SCA uninfected foxes had significantly higher IgG levels than SCZ uninfected foxes at t_1_ (p < 0.001); however, IgG levels were similar at t_2_ (p = 0.1) and t_3_ (p = 0.9). In contrast, SCA infected foxes maintained IgG levels that were significantly higher than SCZ foxes at all recapture events (t_1_ and t_2_: p < 0.001, t_3_: p = 0.001). IgE levels for SCA and SCZ foxes were below the detection limit of the assay; therefore, data were not analyzed further.

### Ear canal tumor occurrence

The initial tumor prevalence in the entire study population of SCA foxes was 21% (23/111). Six foxes were excluded from this analysis due to insufficient biopsies collected at the beginning of the study. The vast majority of tumors at t_0_ were detected in mature foxes, where 51% (22/43) of foxes ≥ 4 years old exhibited ear canal tumors. Although 79% (88/111) of the foxes were initially without tumors, a substantial number of these foxes developed new tumors over the six month trial. During the final capture period (t_3_), 53 of the initially tumor –free foxes were trapped and examined, and 12 of these foxes were ≥ 4 years old. New ear canal tumors were observed in five foxes (5/53), three of which were infected (all untreated) and two of which were uninfected (one treated, one untreated). Again, the majority of tumors were detected in mature foxes, where 33% (4/12) of foxes ≥ 4 years old exhibited new ear canal tumors.

## Discussion

The results of this study provide compelling evidence that acaricide treatment is an effective means of reducing ear mites, and that mite removal reduces known risk factors for ceruminous gland tumor formation in SCA foxes. Mite removal appears to decrease chronic ear canal irritation and excessive cerumen production, likely diminishing pathologic changes in the ear that might lead to tumor formation. The fact that CGH actually increased in such a short period of time within persistently infected foxes provides strong evidence that the long-term presence of mites is likely associated with epithelial hyperplasia. Rapid cell division over time increases the chance of acquiring an oncogenic mutation, leading to uncontrolled growth and the development of epithelial tumors like ceruminous adenomas and carcinomas [22]. Without intervention, hyperplasia in the ear canal will continue to advance in mite-infected foxes, likely increasing the risk of tumor development.

The decrease in mite prevalence and intensity of infection supports the continued use of ivermectin or other acaricides aurally to reduce ear mite infections in SCA foxes. A small number of foxes in the treated group were infected with mites at t_3_; this may be attributable to incomplete elimination of initial infection, or reinfection. Mite count decreased in both treated and untreated groups over the six month study, and although the decrease was much more substantial in the treated group, the mite reduction within the untreated group was unexpected. We suspect that mechanical removal of mites and alteration of the aural environment during sampling and biopsy procedures might have contributed to this overall decline. Administration of lidocaine and application of styptic powder to the biopsy sites, as well as exudate or serum produced by the healing process, may have contributed to a temporary reduction in mite burdens in both groups immediately after biopsy. Any process that increases inflammation and exudate in the ear canal can create an inhospitable environment for ear mites, often causing mites to abandon the ear canal or die, thus decreasing overall mite burden [12]. This “washout effect” of serum or exudate has also been suggested in SCA foxes with severely diseased ear canals where few mites are present [10]. It is also possible that by reducing the mite burden in a proportion of the population, we decreased the rate of reinfection, which occurs almost exclusively through direct contact with infected foxes [12]. Seasonal change in mite burden is unlikely because SCA island’s climate is dry and warm, with only mild fluctuations in temperature and rainfall [23], *O*. *cynotis* is an obligatory ectoparasite that completes all life stages on its host [24], high mite prevalence has been detected in SCA foxes throughout the year, and overall fox body condition does not appear to change with the seasons (JKL, personal communication).

Currently, Island foxes are an intensively managed species, and will be for the foreseeable future. During annual island-wide census trapping on SCA island, a substantial number of foxes are examined, identified with a passive integrated transponder tag, and vaccinated against canine distemper and rabies. If acaricide administration is included in annual trapping efforts, a proportion of the population could be treated regularly, which could result in lower mite prevalence in the population over time. Future longitudinal studies are needed to provide follow-up on individual foxes and their offspring, as well as population-level effects of treatment.

The lack of demonstrable mite-specific IgE in SCA foxes included in this study makes it highly unlikely that an IgE mediated hypersensitivity to mite allergens is involved in the ear pathology observed in mite infected foxes. In contrast, mite-specific IgG was readily demonstrable in the sera of all foxes tested. Although the IgG ELISA results were not significantly different between treated and untreated foxes, both groups demonstrated a decrease in IgG levels between t_0_ and t_3_. This decline in IgG antibodies is likely attributable to the overall decrease in mite count in both groups. Consistent with this observation, we found that uninfected foxes had significantly lower IgG levels than infected foxes. In fact, the levels of IgG in SCA treated and uninfected foxes were similar to that of SCZ foxes that have never been infected with ear mites. While SCZ foxes do not have ear mites, they are expected to have low levels of demonstrable IgG because of potential cross-reactivity with other ectoparasites such as fleas and ticks, internal parasites, or similar environmental antigens. It appears that mite specific IgG antibodies are induced following mite infection and the level of IgG is related to the mite burden within the ear canal. Because IgG can be associated with immune-complex formation and type III hypersensitivity reactions, it is possible that these types of inflammatory responses may exacerbate otitis and CGH [25]. Further investigations to characterize the immunomodulatory effect of mites [26, 27] should reveal any link between IgG antibodies and ear pathology evident in SCA foxes.

The prevalence of tumors at the beginning of the study was 51% among mature foxes. This prevalence is similar to the 52% prevalence of tumors detected from 2007–2008 in Vickers et al. 2015 [10]. The incidence of new tumors during the six month trial was quite high: 33% among mature foxes and 9% across both young and mature age groups. The high tumor incidence emphasizes the need to continue studying this unusual disease process in this endangered population, and developing and testing treatment strategies.

These results advance our understanding of the underlying pathogenesis which results in ceruminous gland tumors. In this trial, we treated ear mites with acaricide and documented its effect on ear canal lesions, including tumor presence. Our findings have helped inform management decisions and now all SCA foxes caught during annual trapping efforts are treated aurally with ivermectin. As a consequence, prevalence of ear mites in the fox population appears to have decreased to approximately 19% (65/340 foxes examined) as of 2014, and treated adults appear to raise uninfected pups more often than untreated adults (JLK, personal communication).

This field trial is novel in that a treatment strategy that may reduce the incidence of cancer was successfully tested in an endangered free-ranging population. The disease threat that ear canal tumors pose in this conservation-dependent species warranted such an intervention, and additional studies, especially those focusing on genetic susceptibility [10], should be conducted to determine why SCA foxes appear to be uniquely susceptible to tumor formation. Many subspecies of Channel Island foxes are already threatened by their genetic and geographic isolation, increasing the risk of infection by various pathogens [8,9,28]. High ectoparasite burdens and cancer are emerging conservation concerns for many wildlife species, and heavy ear mite infections have been documented in several wild canids and felids, and is often associated with population stress [29–34]. The impact of neoplasia on threatened, protected, and endangered species is a growing threat to wildlife survival and conservation [35–39]. The risk factors, pathogeneses, and etiologies contributing to cancer development in wildlife are highly diverse and complex, and there is a strong need for increased research to improve our understanding of infectious and non-infectious tumorigenesis in wildlife populations. This study, and that of Vickers et al. [10], provide an outstanding example of how pathology, epidemiology, disease ecology, and adaptive management can be combined to understand and manage disease in wild populations.

## Supporting Information

S1 Table Individual SCA fox capture and treatment histories and results of otitis, mite, CGH, IgG, and tumor assessments. Otitis and CGH grades are 0–3 and are averaged for both ears, as are mite counts. IgG values are as described in the manuscript. Tumor detection was based on samples obtained during the study. Tumor group (Y/N) denotes individuals whose tumors were detected either during the study or previously.
(PDF)

S1 Fig Summary of capture histories, capture timelines, and mite prevalence among Santa Catalina Island treated foxes. Foxes are divided into subsets (n = 7 horizontal timelines) based on their total number of captures (2 captures = blue dots, 3 captures = green dots, or 4 captures = red dots) and capture intervals (2 months, 4 months, or 6 months). The total number of foxes (n) at each capture period, mite prevalence at that time point, and recapture success is displayed at the bottom of the figure beneath each month. Note: 6 of the 59 treated foxes were never recaptured after t_0_.
(PDF)

S2 Fig Summary of capture histories, capture timelines, and mite prevalence among Santa Catalina Island untreated foxes. Foxes are divided into subsets (n = 7 horizontal timelines) based on their total number of captures (2 captures = blue dots, 3 captures = green dots, or 4 captures = red dots) and capture intervals (2 months, 4 months, or 6 months). The total number of foxes (n) at each capture period, mite prevalence at that time point, and recapture success is displayed at the bottom of the figure beneath each month. *Note: 1 of the 58 untreated foxes in the 6^th^ subset was accidentally treated at t_2_; therefore, it was excluded from the mite prevalence calculation at t_3_ for the 6^th^ subset. Note: 5 of the 58 untreated foxes were never recaptured after t_0_.
(PDF)
