# Supplementary material for: Ear Mite Removal in the Santa Catalina Island Fox (Urocyon littoralis catalinae): Controlling Risk Factors for Cancer Development
Source: PLoS One. 2015 Dec 7;10(12):e0144271. doi: 10.1371/journal.pone.0144271 (PMC4671584; doi:10.1371/journal.pone.0144271)
Supplement: S1 Table — Otitis and CGH grades are 0–3 and are averaged for both ears, as are mite counts. IgG values are as described in the manuscript. Tumor detection was based on samples obtained during the study. Tumor group (Y/N) denotes individuals whose tumors were detected either during the study or previously. (PDF) [file pone.0144271.s001.pdf]

S1 Table

FoxID	Tmt	Otitis t0	Otitis t3	CGH t0	CGH t3	Mites t0	Mites t1	Mites t2	Mites t3	Mite Ct t0	Mite Ct t1	Mite Ct t2	Mite Ct t3	IgG t0	IgG t1	IgG t2	IgG t3	tumor_detected t0	tumor_group t0	tumor_detected t3	tumor_group t3
01A24	N	2.5		1		Y		Y		104		102		115650		76950		N	N		
02056	N	1.5		1.5		Y	Y			64	11			223875	171000			N	N		
05D1D	N	2.5	2	2.5	3	Y		Y	Y	131		197	81	110250		69900	61500	N	N	N	N
07447	N	2.5	2	1.5	2	Y	Y	Y	Y	116	51	108	43	183150	262800	76050	361800	N	N	N	N
07B04	N	2.5		3		Y	Y			9	14			476550	479250			N	N		
13F03	N	3		3		Y	Y			32	29			561600	254475			N	N		
16C09	N	1	1.5	1.5	2.5	Y	Y	Y	Y	57	73	130	140.5	.	119700	64350	49050	N	N	N	N
2024C	N	3	1	3	2	Y	Y		Y	123	58		49	286425	346950		140850	N	N	N	N
22241	N	1.5		1.5		Y	Y	Y		29	9	55		215550	195300	179100		N	N		
25D35	N	2.5	3	1.5	3	Y	Y	Y	Y	130	65	168	98	484650	249750	284400	281925	N	N	N	N
31151	N	3	1.5	2	2	Y		Y	Y	264		265	382	144900		99450	62700	N	N	N	N
3173F	N	0.5	2	1	3	Y	Y	Y	Y	0	4	14	0	92700	103950	119700	72150	Y	Y	N	Y
31E51	N	1.5	0.5	1.5	1.5	Y			Y	290			47	171900			191700	N	N	N	N
33675	N	1.5	2	2.5	3	Y	Y	Y	Y	106	22	38	38	1482300	1372950	1113750	1158300	N	N	Y	Y
34661	N	2	2	2.5	2.5	Y			Y	77			3.5	359100			280125	N	N	N	N
34A46	N	.	2	.	2.5	Y	Y		Y	0	25		67	133650	131400		264375	N	N	Y	Y
34F48	N	3		2		Y				224				299700				N	N		
37734	N	0.5	1.5	1	2	Y	Y	Y	Y	156	31	38	86	572400	577800	545400	487350	N	N	N	N
44D39	N	1	1	1.5	2	Y			Y	65			115	66300			65550	N	N	N	N
5317D	N	3	2	3	3	Y	Y		Y	72	30		15	1449900	1190700		375300	N	N	N	N
54058	N	1		2		Y				102				115200				N	N		
54E06	N	1.5	2.5	1.5	3	Y	Y		Y	1	1		35	388800	390150		465750	N	N	Y	Y
6367D	N	2.5		2.5		Y				110				92625				N	N		
66614	N	0.5	0.5	1	1.5	Y	N	N	N	1	0	0	0	49800	50850	32250	42600	N	N	N	N
74A72	N	2	0.5	2.5	3	Y	Y	Y	N	95	67	13	0	2745900	3402000	1632150	506250	N	N	Y	Y
75152	N	2.5	1.5	2	2.5	Y	Y	Y	Y	75	49	68	104	1287900	452250	411750	476550	N	N	N	N
76509	N	0	1	1	2	Y	Y	Y	Y	16	13	24	9	156600	187650	146700	39900	N	N	N	N
80714	N	1		0.5		Y		Y		332		123		148500		173250		N	N		
81515	N	3		3		Y	Y			62	15			89325	66300			Y	Y		
86116	N	1	0	1	1	Y	N	N	N	1	0	0	0	94950	77475	75675	64500	N	N	N	N
93E02	N	1.5		.		Y	Y	Y		65	77	92		378000	280125	373950		N	N		
95E52	N	1.5	.	1.5	.	Y	Y		Y	134	22		37	120600	86550		55650	N	N	N	N
97589	N	2.5	1.5	2.5	2	Y	Y	Y	Y	496	264	365	141	185850	241875	132750	122400	N	N	N	N
A2E2E	N	2		1.5		Y	Y			27	42			1413450	2549475			N	N		
A370E	N	2.5	2.5	3	3	Y	Y	Y	Y	142	66	118	124	2531250	1688850	1251450	1243350	N	N	N	N
A4C57	N	3		3		Y	Y	Y		137	23	74		144000	90525	494100		N	N		

B637F	N	2.5	1.5	2.5	2.5	Y	Y	Y	Y	10	24	20	10	479250	441450	372600	161550	N	N	N	N
B6972	N	2		1.5		Y	Y			126	74			148950	262125			N	N		
C0C2B	N	2	1	1	2.5	Y	Y		Y	76	35		27	461700	280350		133650	N	N	N	N
C0F5E	N	1	0.5	1	1.5	Y	.		N	21	.		0	122400	63000		35700	N	Y	N	Y
C2451	N	1	1	1	2	Y	Y	Y	Y	479	1	131	44	198000	120600	117450	122400	N	N	N	N
C3D45	N	2		2		Y	Y	Y		77	104	104		967950	939600	815400		N	N		
C476E	N	3	3	3	2.5	Y		Y	Y	107		31	32	468450		652050	351000	N	N	N	N
C4B48	N	0.5	1	2.5	1	Y		N	N	26		0	0	174600		56550	61500	N	N	Y	Y
D030C	N	1.5		1.5		Y	Y	Y		66	12	132		351000	256725	413100		N	N		
D137F	N	2.5	2.5	2.5	3	Y	Y	Y	Y	25	25	85	62	303750	189450	167400	123300	Y	Y	N	Y
D4735	N	1.5		0.5		Y				137				90675				Y	Y		
E0920	N	3	2	3	3	Y			Y	189			238	144000			99750	Y	Y	N	Y
E2E62	N	1	2.5	1.5	3	Y	Y	Y	Y	239	20	54	103	133200	161550	70500	46650	N	N	Y	Y
E6631	N	2		1		Y	Y	Y		35	5	1		517050	209700	162000		N	N		
E7404	N	2.5		2		Y	Y	Y		330	179	208		177300	95625	140400		N	N		
E7700	N	1	2	0	2	Y	Y	Y	Y	0	109	51	2	70500	176400	131400	78563	N	N	N	N
E7F5F	N	2		2		Y				26				183600				Y	Y		
F0255	N	.	0	.	0	Y	N	Y	N	1	0	2	0	62550	71550	67950	29400	Y	Y	N	Y
F0B5E	N	3	1.5	2.5	3	Y	Y	Y	Y	23	11	8	24	115200	136350	81525	46350	N	N	N	N
F1A06	N	1.5	2.5	1.5	2	Y	Y	Y	Y	28	14	3	17	572400	256275	402300	251100	N	N	N	N
F2016	N	2	2	1	3	Y	Y	Y	Y	20	89	60	6	3183300	1275750	2581875	767475	N	N	N	N
F7968	N	0.5		0.5		Y	Y	Y		3	4	2		41100	48000	40500		N	Y		
00241	Y	3	1	3	1.5	Y	Y		N	39	98		0	1174500	1069200		317475	N	N	N	N
00956	Y	1.5	0	1.5	1	Y	N	.	N	88	0	.	0	131400	51750	37350	15150	N	N	Y	Y
01760	Y	1.5		1.5		Y	N			69	0			211050	153450			N	N		
01D42	Y	1.5	0.5	2.5	1	Y	Y	N	N	62	0	0	0	1024650	649350	490050	265950	N	Y	N	Y
03022	Y	2		2		Y		N		18		0		225225		67050		N	N		
05A2A	Y	1.5		1.5		Y	N			27	0			106650	86775			N	N		
07351	Y	2		1.5		Y				10				258075				N	N		
1121D	Y	1.5	0.5	1.5	2	Y	N		N	0	0		0	70950	65550		33000	Y	Y	N	Y
1220F	Y	1.5	0.5	2	1.5	Y			N	135			0	133650			46200	N	N	N	N
23E3C	Y	1	1	2.5	1	Y	N	N	N	127	0	0	0	1040850	234450	150750	131400	N	N	N	N
31A13	Y	2		2.5		Y	N			51	0			1215000	698625			N	N		
32160	Y	3	1	3	1	Y	N	Y	N	78	0	1	0	1988550	.	170100	144000	Y	Y	Y	Y
34237	Y	0.5	0	0.5	0	Y	N	N	N	274	0	0	0	190350	140850	116100	81525	N	N	N	N
34653	Y	1	1	2.5	2	Y	N	N	N	3	0	0	0	340200	263025	207000	163800	Y	Y	N	Y
3487F	Y	1.5	1	1.5	1	Y	N		N	160	0		0	486000	118800		70950	N	N	N	N
36E18	Y	3	0.5	2.5	0.5	Y			N	250			0	162000			85800	N	N	N	N

3795A	Y	0.5		0		Y	N			0	0			103050	130950			N	N		
43A70	Y	2	0	2.5	0.5	Y	Y	N	N	50	1	0	0	1773900	1190700	519750	237038	N	N	N	N
46F1D	Y	0.5	1.5	1.5	3	Y	Y	N	N	1	2	0	0	674325	558900	395550	297225	Y	Y	Y	Y
56174	Y	2.5		2		Y	N			23	0			518400	165600			N	N		
5626F	Y	1.5	0	2	0.5	Y	N		N	182	0		0	192150	84975		81075	N	N	N	N
57B2A	Y	1		1.5		Y	N			186	0			154800	86550			N	N		
61807	Y	2	0.5	2	1.5	Y	Y	N	N	40	1	0	0	392850	169200	126000	41100	N	N	N	N
62023	Y	1	0.5	1	1	Y	N	N	N	47	0	0	0	211500	84000	59100	44550	Y	Y	N	Y
63E72	Y	1.5	1	1.5	2.5	Y		N	N	37		0	0	85800		34500	36075	Y	Y	Y	Y
6585A	Y	0	0.5	0.5	2	Y	N	N	N	2	0	0	0	115200	90675	56400	63000	N	N	N	N
66E41	Y	0.5		2		Y				66				171000				N	N		
76E23	Y	2	1	2	1.5	Y	Y		N	42	5		0	88575	136350		72600	Y	Y	Y	Y
80632	Y	3		2.5		Y				9				491400				N	N		
8201C	Y	1	0.5	1	0	Y	Y		N	45	1		0	72600	73500		29550	N	N	N	N
82954	Y	.		.		Y	N			95	0			797175	591300			N	N		
83364	Y	1.5	.	1.5	.	Y		Y	N	13		15	0	1377000		472500	296550	Y	Y	Y	Y
8430C	Y	3	1.5	2.5	1.5	Y		N	N	16		0	0	618300		224550	165150	N	N	N	N
87861	Y	0.5	2	1	3	Y		N	Y	8		0	29	130050		54300	52350	Y	Y	Y	Y
91B39	Y	3	1.5	2.5	2	Y			Y	97			99	387450			67350	N	N	N	N
92C2D	Y	1.5	1	2.5	1.5	Y			Y	5			1	332100			51000	Y	Y	N	Y
94F6D	Y	3	0.5	3	1.5	Y	N	N	N	49	0	0	0	1279800	461700	345600	270675	N	N	N	N
97F03	Y	1.5		1.5		Y				108				116550				N	N		
A0D0E	Y	1	0	1.5	0	Y	N	N	N	18	0	0	0	71550	72750	67800	57600	N	N	N	N
A1859	Y	1		1		Y	N			48	0			356400	140400			N	N		
A792E	Y	2		2		Y	N	N		111	0	0		255600	74100	96975		N	N		
B1248	Y	2.5	1	3	1	Y			N	52			0	211500			66000	Y	Y	N	Y
B3D37	Y	2	0	2	1	Y	N	N	N	127	0	0	0	246825	112950	60000	55350	N	N	N	N
B7A7B	Y	2.5	0.5	2	1.5	Y	N		N	159	0		0	156600	93975		64650	N	N	N	N
C1B4D	Y	2.5		2.5		Y				14				168300				N	N		
C432B	Y	3	1	2.5	1	Y		N	N	113		0	0	156600		61500	54750	N	N	N	N
C5265	Y	0.5	0	1	1	Y	N	N	N	37	0	0	0	123300	65100	39150	47700	N	N	N	N
C6552	Y	2.5	0.5	2.5	2	Y	Y		N	7	2		0	829575	486000		176850	Y	Y	N	Y
C6553	Y	1.5		2		Y	N			185	0			198000	114975			N	N		
C7646	Y	2.5	1	2.5	3	Y			N	16			0	140400			62850	Y	Y	Y	Y
D1E6A	Y	1.5		0.5		Y				192				338850				N	N		
D3707	Y	.	0	.	1	Y	N		N	15	0		0	521100	162450		70800	N	N	N	N
D3B78	Y	2		2.5		Y	N			292	0			172800	49800			N	N		
D4B71	Y	.	0	.	0	Y	N	N	N	7	0	0	0	410400	246600	98025	61200	N	N	N	N

E1417	Y	2.5	0.5	1.5	0.5	Y	Y		N	72	15		0	205200	139050		57450	N	N	N	N
E2F58	Y	1.5	1.5	0	1.5	Y			Y	40			122	346950			388800	N	N	N	N
E3F49	Y	1.5	1	2.5	1	Y	N		N	77	0		0	379350	92325		42150	N	N	N	N
F1E08	Y	2.5	1	3	1	Y	Y	N	N	13	28	0	0	1421550	865350	399600	268650	N	N	N	N
F452D	Y	2.5		3		Y		N		18		0		399600		137700		N	N		
