# Supplementary material for: Ear Mite Removal in the Santa Catalina Island Fox (Urocyon littoralis catalinae): Controlling Risk Factors for Cancer Development
Source: PLoS One. 2015 Dec 7;10(12):e0144271. doi: 10.1371/journal.pone.0144271 (PMC4671584; doi:10.1371/journal.pone.0144271)
Supplement: S1 Fig — Foxes are divided into subsets (n = 7 horizontal timelines) based on their total number of captures (2 captures = blue dots, 3 captures = green dots, or 4 captures = red dots) and capture intervals (2 months, 4 months, or 6 months). The total number of foxes (n) at each capture period, mite prevalence at that time point, and recapture success is displayed at the bottom of the figure beneath each month. Note: 6 of the 59 treated foxes were never recaptured after t0. (PDF) [file pone.0144271.s002.pdf]

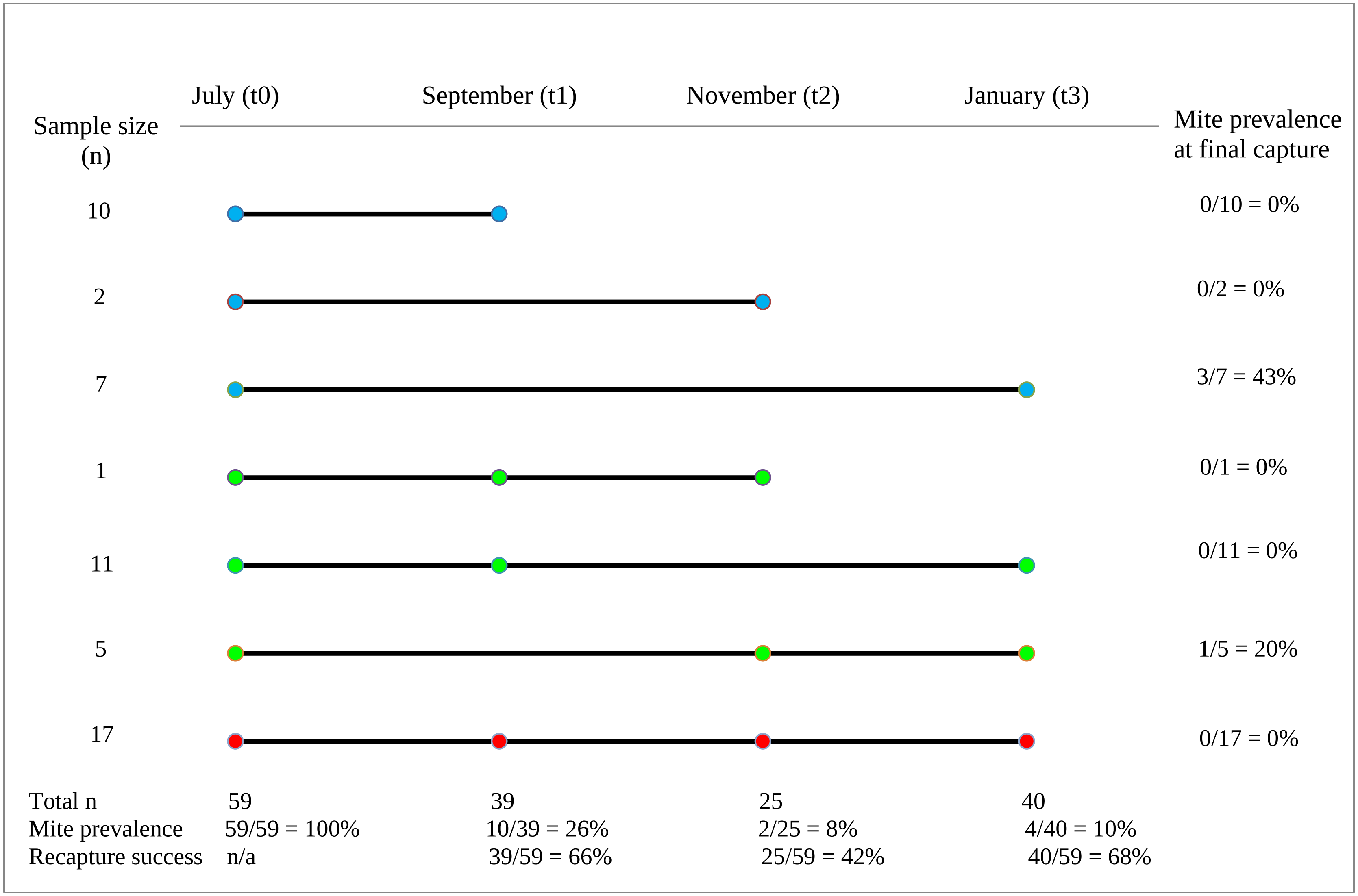

S1 Figure. **Summary of capture histories, capture timelines, and mite prevalence among Santa Catalina Island treated foxes**.

Foxes are divided into subsets (n = 7 horizontal timelines) based on their total number of captures (2 captures = blue dots, 3 captures = green dots, or 4 captures = red dots) and capture intervals (2 months, 4 months, or 6 months). The total number of foxes (n) at each capture period, mite prevalence at that time point, and recapture success is displayed at the bottom of the figure beneath each month. Note: 6 of the 59 treated foxes were never recaptured after t_0.
