# Supplementary material for: Ear Mite Removal in the Santa Catalina Island Fox (Urocyon littoralis catalinae): Controlling Risk Factors for Cancer Development
Source: PLoS One. 2015 Dec 7;10(12):e0144271. doi: 10.1371/journal.pone.0144271 (PMC4671584; doi:10.1371/journal.pone.0144271)
Supplement: S2 Fig — Foxes are divided into subsets (n = 7 horizontal timelines) based on their total number of captures (2 captures = blue dots, 3 captures = green dots, or 4 captures = red dots) and capture intervals (2 months, 4 months, or 6 months). The total number of foxes (n) at each capture period, mite prevalence at that time point, and recapture success is displayed at the bottom of the figure beneath each month. *Note: 1 of the 58 untreated foxes in the 6th subset was accidentally treated at t2; therefore, it was excluded from the mite prevalence calculation at t3 for the 6th subset. Note: 5 of the 58 untreated foxes were never recaptured after t0. (PDF) [file pone.0144271.s003.pdf]

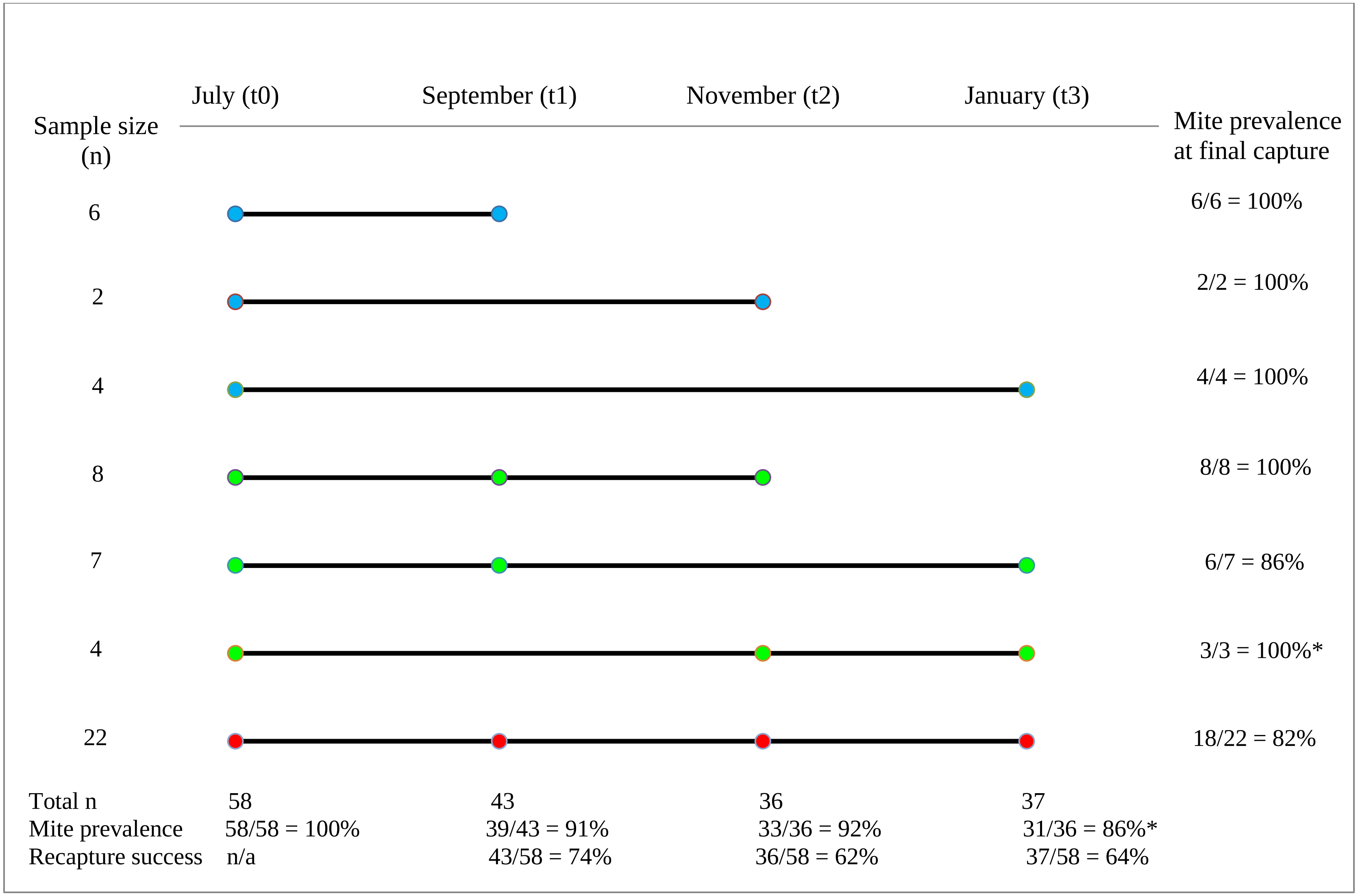

S2 Figure. **Summary of capture histories, capture timelines, and mite prevalence among Santa Catalina Island untreated foxes**.

Foxes are divided into subsets (n = 7 horizontal timelines) based on their total number of captures (2 captures = blue dots, 3 captures = green dots, or 4 captures = red dots) and capture intervals (2 months, 4 months, or 6 months). The total number of foxes (n) at each capture period, mite prevalence at that time point, and recapture success is displayed at the bottom of the figure beneath each month. *Note: 1 of the 58 untreated foxes in the 6th subset was accidentally treated at t_2; therefore, it was excluded from the mite prevalence calculation at t_3 for the 6th subset. Note: 5 of the 58 untreated foxes were never recaptured after t_0.
